# Supplementary figures and images for: Trpc6 gain-of-function disease mutation enhances phosphatidylserine exposure in murine platelets
Source: PLoS One. 2022 Jun 24;17(6):e0270431. doi: 10.1371/journal.pone.0270431 (PMC9231752; doi:10.1371/journal.pone.0270431)

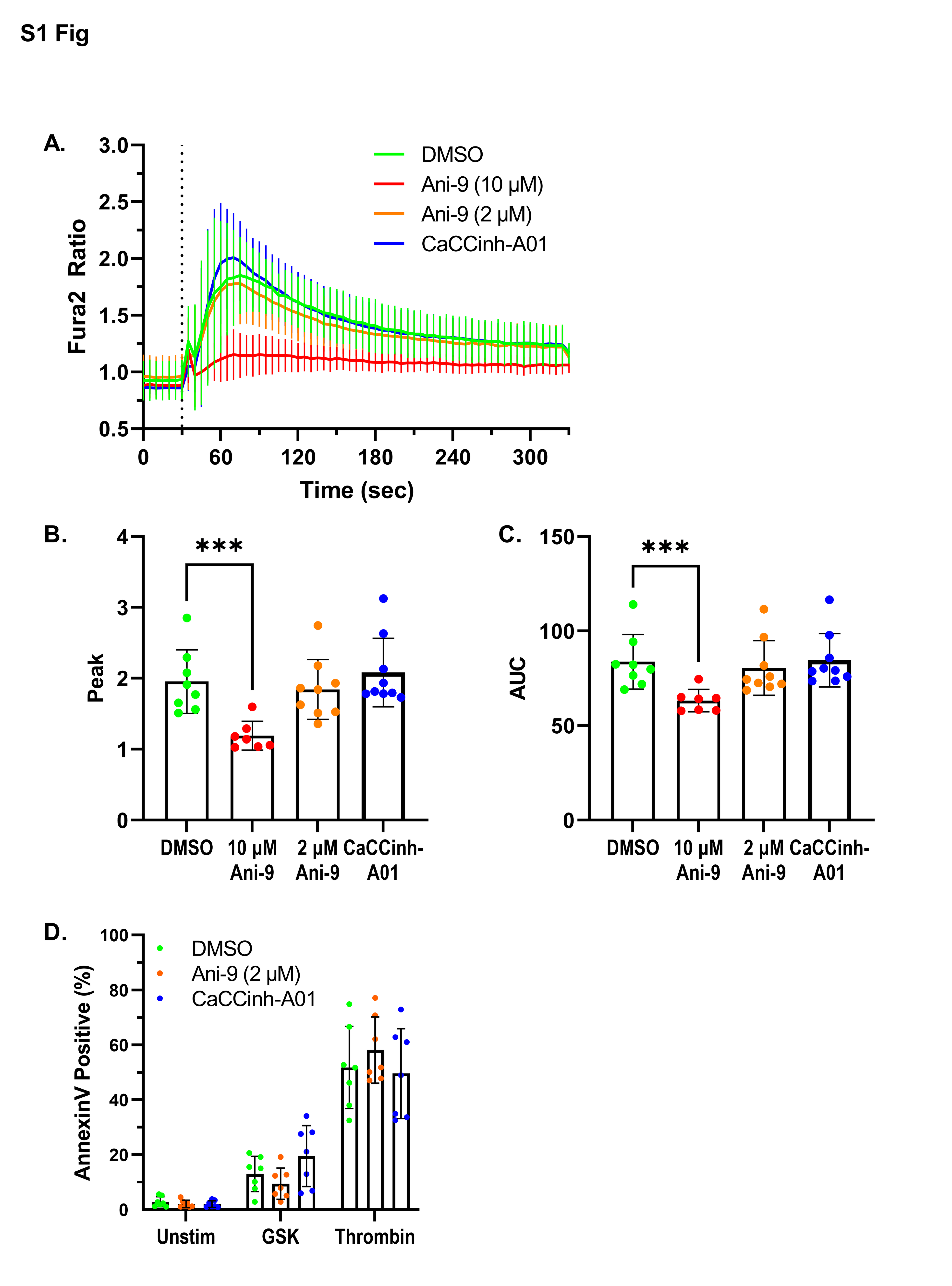

Supplement: S1 Fig — A-C, KI platelets, pre-incubated in the presence of vehicle (DMSO), Ani 9 (2 μM or 10 μM), or CaCCinh-A01 (25 μM) were subject to Fura-2 ratiometric imaging. GSK was added after 30 seconds. A, Fura-2 fluorescence ratio (340/380) time-course; n = 7–9. The post-stimulation Fura-2 fluorescence ratio peak (B), and area under the curve (C), are shown. Mixed-effects analysis with Dunnett’s multiple comparisons test; only statistically significant comparisons between DMSO and inhibitor treated conditions are shown. D, percentage of KI platelets stained for Annexin V. Platelets were pretreated with DMSO, Ani 9 (2 μM), or CaCCinh-A01(25 μM), followed by stimulation with buffer (Unstim), GSK, or thrombin; n = 7. Two-way ANOVA with Dunnett’s multiple comparisons test; no statistically significant pairwise comparisons between DMSO and inhibitor treated conditions. (TIF) [file pone.0270431.s001.tif]
