# Supplementary material for: Trpc6 gain-of-function disease mutation enhances phosphatidylserine exposure in murine platelets
Source: PLoS One. 2022 Jun 24;17(6):e0270431. doi: 10.1371/journal.pone.0270431 (PMC9231752; doi:10.1371/journal.pone.0270431)

Figure 1 Original Blots

Figure 1A top panel (TRPC6 WB)

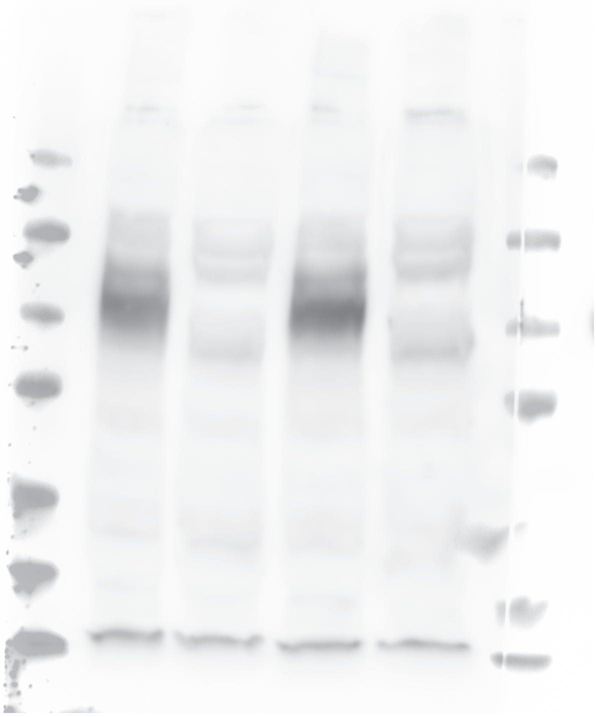

Figure 1A bottom panel (Erk!/2 WB)

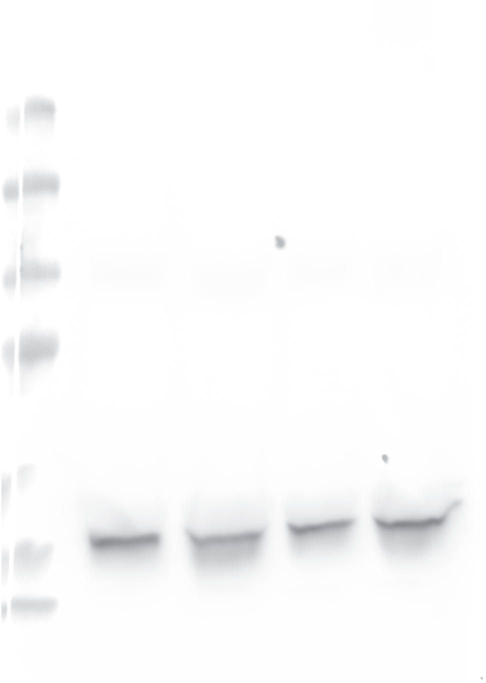

Figure 1B top panel (TRPC6 WB)

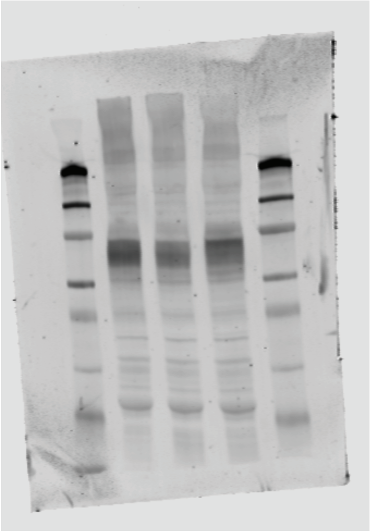

Figure 1B bottom panel (Erk1/2 WB)

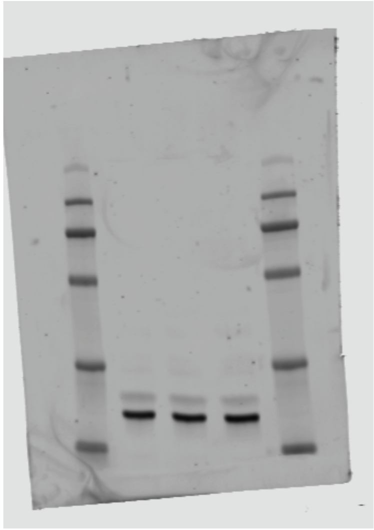

Figure 4 Original Blots

Figure 4A Top panel (P-Erk1/2 Western blot)

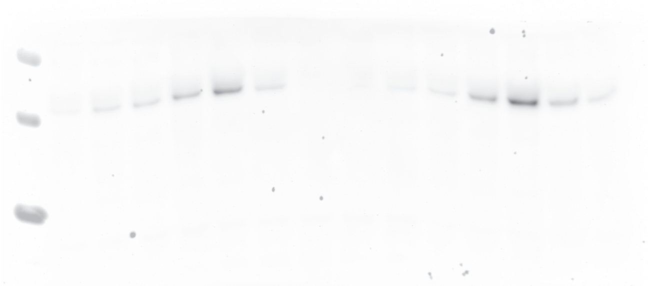

Figure 4A Bottom panel (Erk1/2 Western blot)

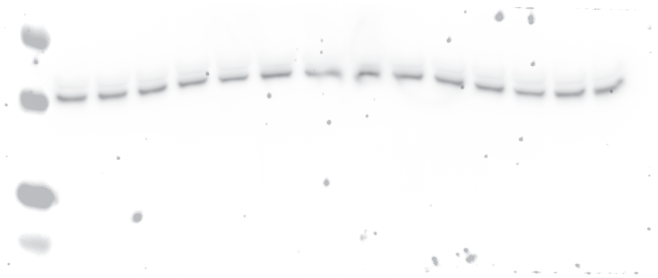

Figure 4D Top panel (P-Erk1/2 Western blot)

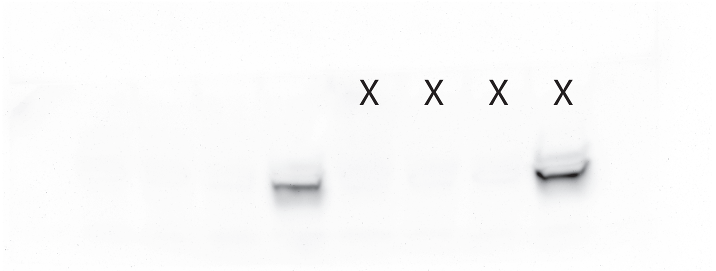

Figure 4D Bottom panel (Erk1/2 Western blot)

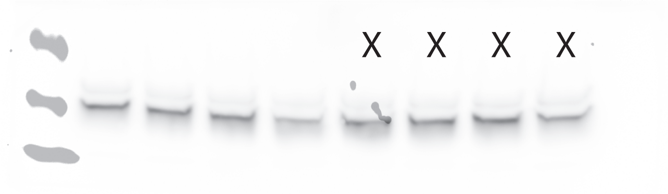

Figure 4B Top panel (P-Erk1/2 Western blot)

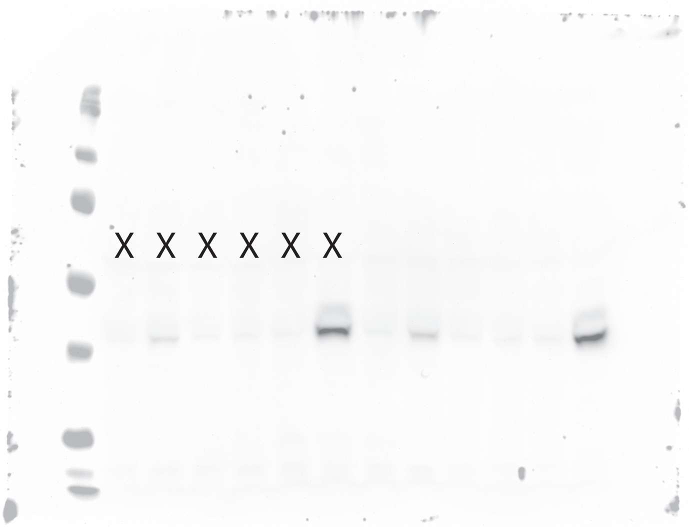

Figure 4B Bottom panel (Erk1/2 Western blot)

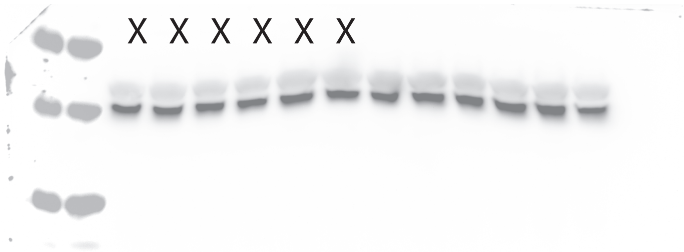

Figure 4E Top panel (P-MPLC2 Western blot)

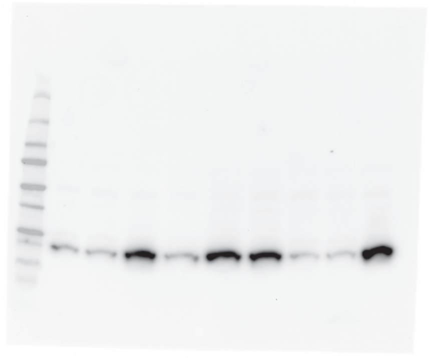

Figure 4E Bottom panel (Actin Western blot)

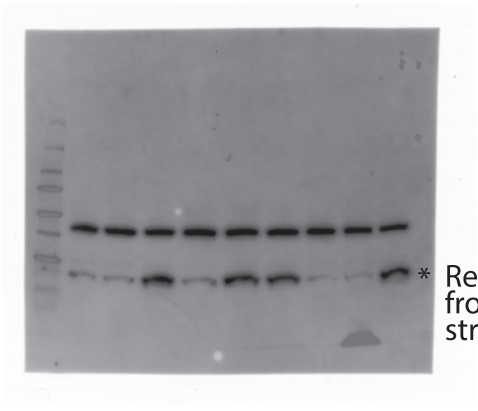

* Residual P-MLC2 signal from incompletely stripped blot

Figure 5 Original blots

Figure 5A Top panel (Talin WB)

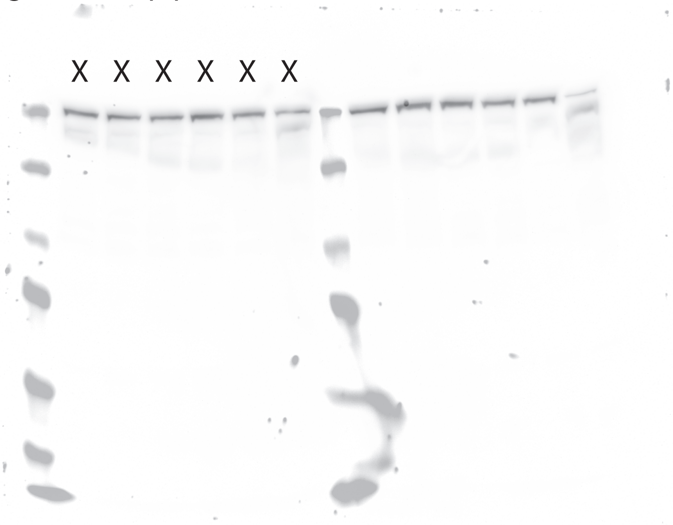

Figure 5A Bottom panel (Erk1/2 WB)

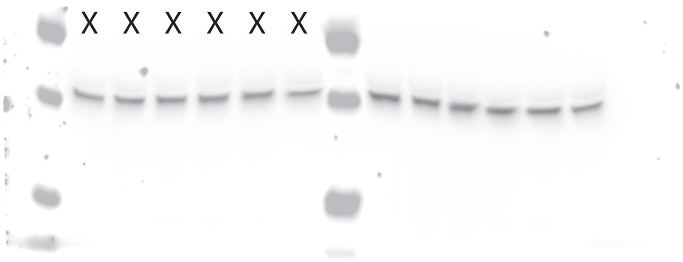

Figure 5B Top panel (Talin WB)

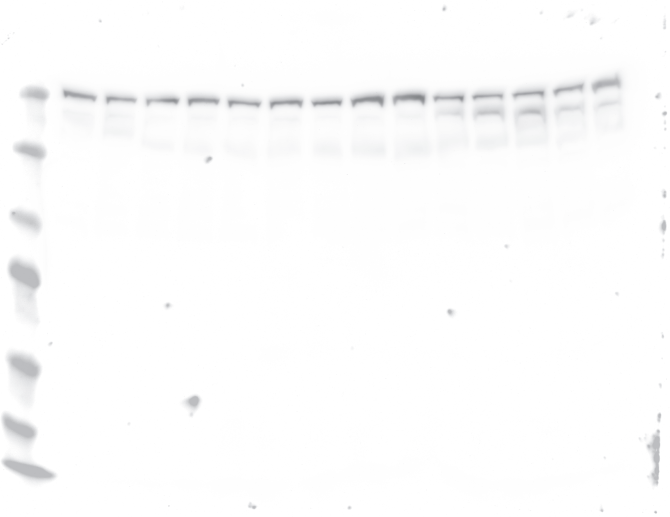

Figure 5B Bottom panel (Actin WB)

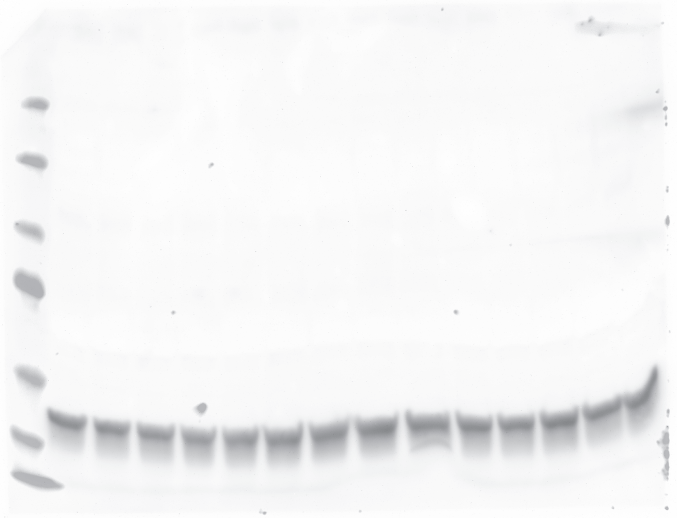

Supplement: S1 Raw images — (PDF) [file pone.0270431.s002.pdf]
